# Learning Attentional Communication with a Common Network for Multiagent Reinforcement Learning

**DOI:** 10.1155/2023/5814420

**Published:** 2023-06-28

**Authors:** Wenwu Yu, Rui Wang, Xiaohui Hu

**Affiliations:** ^1^University of Chinese Academy of Sciences, Beijing 100049, China; ^2^Institute of Software Chinese Academy of Sciences, Beijing 100190, China

## Abstract

For multiagent communication and cooperation tasks in partially observable environments, most of the existing works only use the information contained in hidden layers of a network at the current moment, limiting the source of information. In this paper, we propose a novel algorithm named multiagent attentional communication with the common network (MAACCN), which adds a consensus information module to expand the source of communication information. We regard the best-performing overall network in the historical moment for agents as the common network, and we extract consensus knowledge by leveraging such a network. Especially, we combine current observation information with the consensus knowledge to infer more effective information as input for decision-making through the attention mechanism. Experiments conducted on the StarCraft multiagent challenge (SMAC) demonstrate the effectiveness of MAACCN in comparison to a set of baselines and also reveal that MAACCN can improve performance by more than 20% in a super hard scenario especially.

## 1. Introduction

In real life, multiple autonomous agents need to work together to complete a large number of complex tasks, such as formation control [1], autonomous vehicles [2], unmanned aerial vehicle [3], and multiplayer confrontation games [4]. Multiagent reinforcement learning (MARL) has made significant progress in optimizing the cumulative global rewards in these multiagent systems [5]. However, several open problems are still not well solved in the field of MARL. One of the problems is to learn cooperative behaviours between agents under the partial observation.

For the partially observable multiagent cooperative task, one approach is to use value-based reinforcement learning [6]. Independent Q-learning [7] is a typical representative of applying single-agent reinforcement learning methods directly to multiagent problems. Although independent Q-learning has good scalability, it brings nonstationarity due to the constant changes in the strategies of other agents in the training process, where the performance of each agent is often poor. Based on the series of value decomposition methods [5, 8, 9], the paradigm of the centralized training and decentralized execution (CTDE) [10] is adopted to deal with the situation, in which all agents are centrally controlled during training and each agent individually utilizes a distributed policy during execution. Another way is to exploit the communication in MARL. The CommNet method proposed in [11] employs a continuous communication channel in which an agent gets the sum of the information transmitted by other agents. The communication module is also utilized for engaging with other agents inside policy or critic networks [12]. Typically, agents take the current observation or hidden layer information as the raw information input for communication [13], which limits the source of information.

To address the issues, we add an additional common network for each agent to preserve the historically best-performing overall policy network. The overall policy consists of the policies of all agents. The common network with the best historical performance, which represents the optimal overall policy to a certain extent, can predict the goals to be achieved by future agents. The ultimate goal of training is to obtain the optimal policy, and similarly, the historical optimal overall network can infer the future strategy information of other agents. We name the historical optimal overall network as the common network, and the information obtained from the common network is called consensus information. Significantly, by integrating this consensus information, the communication message can contain the guidance information of the optimal network for the current state in different periods, where an agent can have more raw information to filter, and the common network is screened by the average score of each episode of an agent during the test. Therefore, we integrate the consensus information and observation information, extract the information based on the attention mechanism, and then utilize the extracted information for decision-making to accelerate cooperative learning among agents. Additionally, the common network, being historically best-performing, can be regarded as an expert network to guide the policy search [14]. We exploit the dataset aggregation (DAgger) framework [15], which collects the data from experts and the current policy to enhance the training dataset. Furthermore, we separately assess the viability of the common network as an expert network to improve the performance of agents within the DAgger framework.

In this paper, we add communication channels between each agent to cooperate better. Traditional communication ways, such as directly fixed communication messages, the exchange of discrete information, or a simple summation of consecutive communication messages, are too plain to be used in complex cooperative tasks. The number of communication messages received by agents increases as their number increases, which is not conducive to their decision-making. Therefore, we propose to add communication channels for each agent, allowing the communication information between agents to be extracted by the multihead attention mechanism. The attention-based communication module can better deal with the change in the number of agents.

The proposed approach, which is multiagent attentional communication with the common network (MAACCN) for partially observable cooperation problems, adopts a CTDE framework. The main contributions of the algorithm in this paper are as follows:

1. We add an additional common network to save the historically optimal overall policy for all agents, which gives each agent another source of information to make decisions
2. We process communication and consensus information via the attention mechanism in order to extract more effective information
3. We demonstrate additional experiments in the DAgger framework to verify the feasibility of the common network as an expert network to gather data and improve the performance of agents on the StarCraft multiagent challenge

## 2. Related Work

After reaching outstanding results in the single-agent setting [16], researchers move on to the more challenging multiagent environment [17, 18]. The most straightforward approach to multiagent learning is to have each agent train and learn on their own [19]. This early approach, known as the independent Q-learning (IQL) method, was one of the first of its kind, although it does not generally fare well in practical applications. Despite this, many challenges employ IQL as the baseline for experimental comparison due to the ease of implementation and strong scalability as the number of agents increases.

To further the application of deep deterministic policy gradient (DDPG) to the field of multiagent systems [20], the multiagent DDPG (MADDPG) algorithm was suggested in [21] as part of the research algorithm for multiagent cooperative tasks. In the training phase, MADDPG can obtain the behaviours of all agents, allowing it to solve the problem that each agent in a hybrid environment has its own local reward. Based on MADDPG, the authors of [22] propose shared memory as a form of communication, and a different MADDPG-MD [23] algorithm with improved robustness is inspired by dropout.

Another way to deal with the problem of global reward credit allocation is through value function decomposition. The VDN algorithm presented in [8] decomposes a global Q-function into the sum of the individual local Q-functions of the agent, alleviating the problems of lazy agents and credit allocation among agents. In addition to adding a hybrid network and a nonlinear component to the decomposition on the foundation of VDN, the Q-value mixing network (QMIX) [9] guarantees that the global Q function is monotonous in the local Q function. The COMA [24] algorithm measures the contribution of an agent to the global reward based on the counterfactual baseline. In order to be suited for tasks that satisfy the decomposition conditions but not monotonicity in QMIX, the QTRAN [25] algorithm reduces the structural limitations of QMIX in order to be able to handle more generic problems. Theoretically, Qatten [26] provides a generic decomposition of the value function that explicitly models the influence of intelligence on the whole, based on multihead attention. The regularized softmax (RES) algorithm [27] is an enhancement of QMIX that solves the overestimation of the Q-value, which employs the softmax approach while computing the target.

In terms of multiagent communication, the authors of [28] first introduce communication information into deep reinforcement learning with the goal of resolving the discrete communication channel problem, which combines DQN and IQL applied to multiagent problems. The CommNet algorithm permits multistep communication, and the gradient can be transmitted back to each agent via the continuous communication channel. BiCNet [29] presents a bidirectional recurrent network to each agent, which conceals the information exchange between agents. In order for agents to learn to communicate better, the IC3Net proposed in [13] includes a gate control for each agent so that they can decide whether or not to communicate with other agents.

In terms of screening effective information, the attention model has been widely used in computer vision [30], plant species recognition [31], resource allocation [32, 33], and reinforcement learning as a successful method. The ATOC algorithm suggested in [34] gives agents the ability to choose whether or not to contact one another, as well as which other agents they want to connect with. The method of targeted multiagent communication (TarMAC) [12] uses a signature-based soft attention mechanism during several communication rounds to gauge the importance of communication. The G2ANet [35] method employs a two-stage attention network model, using the hard attention mechanism to define the interactive agent and the soft attention mechanism to determine the weight of the interaction, and automatically learns the relationship between the constantly changing agents in large-scale complicated games.

In the field of multiagent modeling, several earlier efforts have learned the models of other agents through observation. The SOM [36] algorithm employs its own approach to forecast the behaviours of adversaries, infers the target information of other agents, and then makes decisions based on this target information. By providing agents with intrinsic rewards that have a causal impact on the behaviour of other agents, the social influence approach [37] seeks to create coordination and communication in MARL through a unified strategy. However, both of these approaches involve extra work to build this predictive network using supervised learning methods.

Most of these communication algorithms are limited to the hidden layer information of the policy network at the current moment. Our MAACCN algorithm expands the source of communication information by incorporating the common network, and the algorithm of MAACCN selects the algorithm flow of the classical value decomposition series as the fundamental framework to improve both the consensus information module and the communication module, which extract the effective communication information for better collaboration among agents, and in contrast to the SOM algorithm, we select a common network with the best historical performance as the expert network without additional training. Additionally, we take advantage of the DAgger framework, which gathers information from experts and current policies to improve the training dataset.

## 3. Background

This section introduces some of the ideas behind the DAgger framework and MARL based on the value-based algorithm.

### 3.1. DAgger

With the DAgger framework, we can run both the expert policy we want to clone and the novice policy we need to teach at the same time, expanding the capabilities of conventional supervised learning techniques [15]. By aggregating additional expert evidence, the reward structure and the fundamental model are revealed.

DAgger collects more training instances from a combination of the current policy $\tilde{\pi }$ and the expert policy *π*^*∗*^ in an iterative manner. A decision rule governs the interaction of the current policy and the combined expert with the environment during a certain episode, following the new policy:

(1) $$\begin{array}{c} \pi_{\theta_{i}}=\alpha_{i}\pi^{*}+\left(1-\alpha_{i}\right)\tilde{\pi }\text{,} \end{array}$$

where *α*_*i*_ is annealed progressively from 1 to 0.1. DAgger takes the new dataset *D* by the new policy *π*_*θ*_*i*__ with each iteration to retrain the next policy as follows:

(2) $$\begin{array}{c} D\leftarrow \tilde{D}\cup D_{i}\text{,} \end{array}$$

where *D*_*i*_ is gathered by the expert policy *π*^*∗*^ and $\tilde{D}$ is the previous set.

### 3.2. Reinforcement Learning

MARL involves many agents and numerous states, which is the combination of the Markov decision process and the matrix game, whereas the Markov decision process involves one agent and multiple states and the matrix game involves multiple agents and one state. The evolution of MARL is inextricably linked to game theory [38], and the partly observable multiagent cooperation problem (Dec-POMDP) can be characterized as 〈*N*, *𝒮*, *𝒜*, *R*, *P*, *𝒪*, *γ*〉. It is defined for N agents by the global states *𝒮*, action sets *𝒜*={*𝒜*_1_,…, *𝒜*_*N*_}, reward function *R*, environmental state transition function *P* : *𝒮* × *𝒜*_1_ × ⋯×*𝒜*_*N*_⟶*𝒮*, observation space *𝒪*={*𝒪*_1_,…, *𝒪*_*N*_}, and discounted factor *γ*. The learning policy of the agent *i* is *π*_*θ*_*i*__ : *𝒪*_*i*_ × *𝒜*_*i*_⟶[0,1]. At time *t*, the agent *i* receives a reward *r*_*i*_^*t*^ : *𝒮* × *𝒜*_*i*_⟶*R* from the environment after executing action *a*_*i*_^*t*^. In this study, we investigate a purely cooperative task in which each agent receives the same reward, and the overall objective is to discover the most effective cooperative method in order to maximize the cumulative reward *G*=∑_*t*=0_^*T*^*γ*^*t*^*r*^*t*^.

The VDN [8] method is a value decomposition structure based on DRQN [39] to learn the action value function of distinct agents using only global benefits, thereby resolving the issue of partially observable fake gains and lazy agents. The value decomposition function of VDN is as follows:

(3) $$\begin{array}{c} Q\left(\left(h_{1},h_{2},\ldots ,h_{d}\right),\left(a_{1},a_{2},\ldots ,a_{d}\right)\right)\approx \overset{d}{\underset{i=1}{\sum }}{\tilde{Q}}_{i}\left(h_{i},a_{i}\right)\text{.} \end{array}$$

QMIX [9] is an enhanced version of VDN that employs a hybrid network to combine local agent functions and global state information to give positive weights for the hybrid network during training. Taking argmax for the joint action value is the same as taking argmax for each local action value function as follows:

(4) $$\begin{array}{c} {\mathrm{argmax}}_{u}Q_{tot}\left(\tau ,u\right)=\left(\begin{array}{c} {\mathrm{argmax}}_{u_{1}}Q_{1}\left(\tau_{1},u_{1}\right)\\ \vdots \\ {\mathrm{argmax}}_{u_{n}}Q_{n}\left(\tau_{n},u_{n}\right) \end{array}\right)\text{.} \end{array}$$

QMIX converts the preceding expression into a monotonic constraint, which is realized by a hybrid network. The following is the restriction:

(5) $$\begin{array}{c} \frac{\partial Q_{tot}}{\partial Q_{i}}\geq 0,\forall i\in \left\{1,2,\ldots ,n\right\}\text{.} \end{array}$$

## 4. Multiagent Attentional Communication with the Common Network

In order to more effectively handle the problem of multiagent communication and cooperation and assure the adequacy and efficacy of communication information, we propose the multiagent attentional communication with the common network (MAACCN) learning algorithm, which regards the common network as the expert to each agent based on the DAgger framework and employs the attentional mechanism to process the consensus information and communication information. This section elaborates on the concept of our algorithm and the structure before introducing the attention unit and further explores the influence of the common network.

### 4.1. The Framework of MAACCN

In the problem of multiagent cooperation, agents can make better decisions if they can infer the consensus information of other agents based on their states, actions, and thoughts. From this perspective, we suggest the common network with the highest historical performance of agents, from which we obtain the consensus knowledge. More effective information can be gathered through the attention mechanism to extract consensus information, and this part of the information can be incorporated into the policy of each agent, which can enhance the capacity of the agent for decision-making.

**Figure 1:**
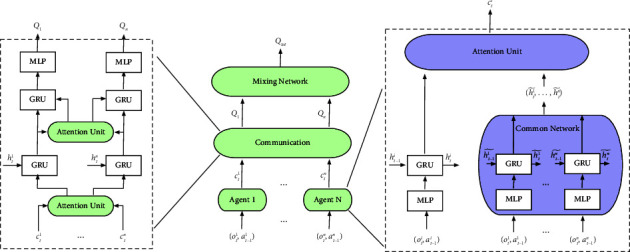
Overall framework of multiagent attentional communication with the common network. Right: the agent *i* first obtains consensus information of other agents from the common network. The feature *h*_*t*_^*i*^ is the hidden layer output of the agent *i* policy at times *t*. The feature *c*_*t*_^*i*^ is the output of the agent *i* through the attention unit at times *t*. Left: the communication module between agents is implemented via the attention mechanism. The feature *h*_*t*_^*n*^ is the hidden layer output of the agent *n* policy at times *t*. The feature *c*_*t*_^*n*^ is the output of the agent *n* through the attention unit at times *t*.

The framework of the proposed multiagent attentional communication with the common network (MAACCN) algorithm is shown in Figure 1, which is divided into three stages. The first stage of MAACCN is the processing of information features, in which the common network can be accessed by each agent. The structure of the common network is the aggregation of the policy networks of all agents. The common network contains policies of all agents. Therefore, inputs of the common network are the observations of all agents. The feature *h*_*t*_^*i*^ is obtained by processing the action-observation(*o*_*t*_^*i*^, *a*_*t*−1_^*i*^) through the gated recurrent unit (GRU) network. The attention mechanism is used to assign weights to the consensus information $\left(\tilde{h_{t}^{1}},\ldots ,\tilde{h_{t}^{n}}\right)$ of other agents collected through the common network in order to generate the output *c*_*t*_^*i*^. The second stage is the communication module, which expands the communication channel among agents based on the multihead attention mechanism. Each agent broadcasts the information to be conveyed and selects the important information received from other agents based on the multihead attention mechanism in order to get the information that is effective for its own decision-making. Before making a decision, an agent engages in numerous iterations of communication with other agents to ensure an adequate exchange of information. The third stage involves integrating the local *Q*_*i*_ function using the mixing network to get *Q*_*tot*_.

In the framework of the method described above, all agents share the same set of network parameters, and different types of information can be acquired based on the observations and the ID numbers of agents at different times. Therefore, the historically optimal policy of each agent is the same. In order to alleviate the challenges caused by partial observability, actions and communication information are passed to agents at the next moment. In the second stage, only one GRU neural network can be utilized for cyclic and iterative communication since the two GRU neural networks that the communication module needs to transit through can share parameters.

The algorithm of MAACCN utilizes offline updating to add the state, action, reward, and termination state of numerous agents interacting with the environment to the experience pool, where a batch of complete episodes is selected for learning. Similar to DQN, our algorithm constructs a target network and duplicates the current network parameters every fixed steps to calculate the value of the next moment, which can expedite convergence and contribute to the stability of the algorithm. The loss function of the algorithm is

(6) $$\begin{array}{c} \begin{array}{c} \text{loss}=\overset{T}{\underset{t=1}{\sum }}{\left(\overset{n}{\underset{i=1}{\sum }}y_{t}^{i}-\overset{n}{\underset{i=1}{\sum }}Q_{i}\left(o_{t}^{i},a_{t}^{i};\theta \right)\right)}^{2}\text{,}\\ y_{t}^{i}=\left\{\begin{array}{c} r,\text{if teminal}\\ r+\gamma \;\max_{a^{\prime }}\;Q^{\text{target}}\left(o_{t+1}^{i},a^{\prime };\theta^{\prime }\right)\text{,} \end{array}\right. \end{array} \end{array}$$

where *θ*′ is a parameter for the target network *Q*^target^. The procedure of MAACCN is described in Algorithm 1 in Appendix, which is a value-based algorithm. In the MAACCN training procedure, the optimal Q-value function estimation is found by minimizing the loss function. During the learning process of agents, the previous common network needs to be replaced by the policy network, which has better performance.

### 4.2. Common Network and Attention Mechanism

Among the many approaches that can be taken to mitigate the problem of instability in a multiagent environment, it is more helpful to model the behaviours of other agents and infer the consensus information of other agents than to simply treat the other agents as part of the environment. Humans use the aims, beliefs, and preferences of other groups with whom they interact to make better judgements, according to cognitive science research. Humans mimic the behaviours of others based on their observations, a cognitive process that enables them to better comprehend the consensus and actions of others and to react appropriately in social circumstances. Inspired by this, we add a common network for all agents.

In contrast to other methods of modeling the aims or actions of other agents, the common network is a replication of the historically optimal overall network, which is judged according to the average score of each episode during the test. Each common network is the same for each agent due to parameter sharing. Thus, an agent can deduce from the common network the consensus information of other agents for communication. In light of one of the fundamental assumptions of the algorithm, namely, that the common network can be used temporarily as the optimal policy network, we investigate the effect of the common network based on the DAgger framework in order to establish the validity of consensus information. Therefore, the policy *π*^*∗*^ of the common network and the current policy $\tilde{\pi }$ jointly decide on the final policy *π*_*θ*_*i*__.

The policy network of each agent receives the observation *o*_*t*_^*i*^, which is then passed through the fully connected layer and RELU function before being input *fc*_*t*_^*i*^ into the GRU cyclic neural network in order to generate the output *h*_*t*_^*i*^. The other input of GRU comes from the output *h*_*t*−1_^*i*^ obtained after processing the information the last time. The formula is as follows:

(7) $$\begin{array}{c} r=\sigma \left(W_{ir}fc_{t}^{i}+b_{ir}+W_{hr}h_{t-1}^{i}+b_{hr}\right)\text{,}\\ z=\sigma \left(W_{iz}fc_{t}^{i}+b_{iz}+W_{hz}h_{t-1}^{i}+b_{hz}\right)\text{,}\\ h_{t-1}^{i^{\prime }}=h_{t-1}^{i}⊙r\text{,}\\ h_{i}^{\prime }=\tan\;h\left(W_{ih}fc1_{t}^{i}+b_{ih}+W_{hh}h_{t-1}^{i^{\prime }}+b_{hh}\right)\text{,}\\ h_{t}^{i}=\left(1-z\right)⊙h_{t-1}^{i}+z⊙h_{i}^{\prime }\text{,} \end{array}$$

where *σ* and tanh are the activation function, *W* and *b* represent the weight matrix and the bias to be trained, and *r* and *z*, respectively, represent reset gates and update memory gates to make more effective use of past data and alleviate local observation constraints.

**Figure 2:**
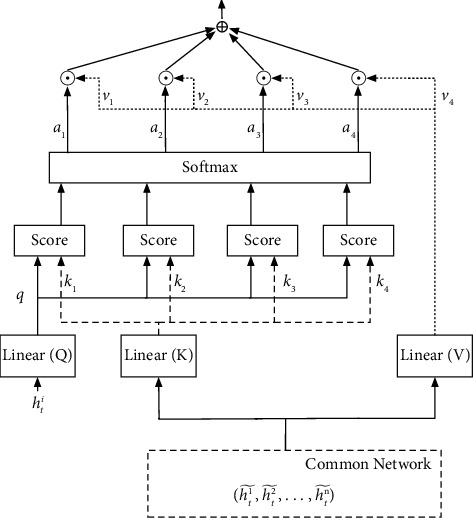
Extracting significant information from the common network based on the multihead attention mechanism, where linear(*Q*) means *Q* is a linear layer and *K* and *V* are also linear layers.

As visualized in Figure 2, the information from these hidden layers is processed using the multihead attention mechanism so that additional valuable information can be collected to help agents cooperate. The attention unit can be depicted as the given information input $X=\left(h_{t}^{i},\tilde{h_{t}^{1}},\ldots ,\tilde{h_{t}^{n}}\right)$, the vector *h*_*t*_^*i*^ being the information of an agent at the current time, and the scoring mechanism $score=key^{T}q/\sqrt{d}$ employing the scaling dot product model, where *d* is a constant. The attention function maps a query and a set of key-value pairs to the following output:

(8) $$\begin{array}{c} \begin{array}{c} q=Qh_{i}^{t}\text{,}\\ key=KX\text{,}\\ value=VX\text{,}\\ h_{i}^{c}=value\times softmax\left(score\right)\text{.} \end{array} \end{array}$$

First, a linear feature transformation is applied to the current vector *h*_*i*_^*t*^, in order to generate a new vector *q*. The *key* and *value* vectors are then obtained following matrix transformations for the input data *X*. The score is then computed, followed by the various weights of the *value*. The final attention information *h*_*i*_^*c*^ is then obtained by multiplying these vectors by the corresponding weights.

## 5. Experiments

This section introduces the experimental setting and assesses the performance of MAACCN, and we describe the SMAC experiment scenarios, experimental parameters settings, and baseline algorithm before analyzing the performance of MAACCN and the ablation experiment findings to verify the effectiveness of the algorithm. Additionally, the viability of the historical optimum network as the common network is examined.

### 5.1. Setting

The gaming settings of SMAC are meticulously crafted so that agents must master one or more micromanagement skills in order to vanquish their adversaries [40]. Each scene involves a clash between two forces, with the initial position, number, and kind of each force varying from scene to scene.

Each agent receives a local observation of its field of vision at each time step, which contains map data within the circular area of each cell. Specifically, the feature vector comprises both friendly and hostile attributes inside the range of view. In this partial observation, an agent is unable to discern whether the remaining agents are out of sight or dead. Following previous work [8, 9], we adopt various hyperparameters. In particular, Table 1 in Appendix contains the algorithm parameters.

### 5.2. Evaluation of MAACCN

The comparison experimental results of the MAACCN algorithm with baseline algorithms in five experimental scenarios of SMAC are first shown in this section, and these results are then further investigated.

The game win rate is chosen as the ultimate evaluation criterion since the goal of these experimental scenarios is to learn how to guide the team under our direction toward success. Every *evaluate*_*cycle* time during algorithm training, the algorithm evaluates the learned policy of an agent. An agent runs *evaluate*_*epoch* rounds of game tests in the associated setting and provides our game victory percentage by tallying the number of victories. To avoid losing generalizability, various random seeds are utilized in the algorithm, four repeated tests are undertaken, and 95% confidence intervals are employed. Following training, statistics of the winning rate are used to draw the curve.

**Figure 3:**
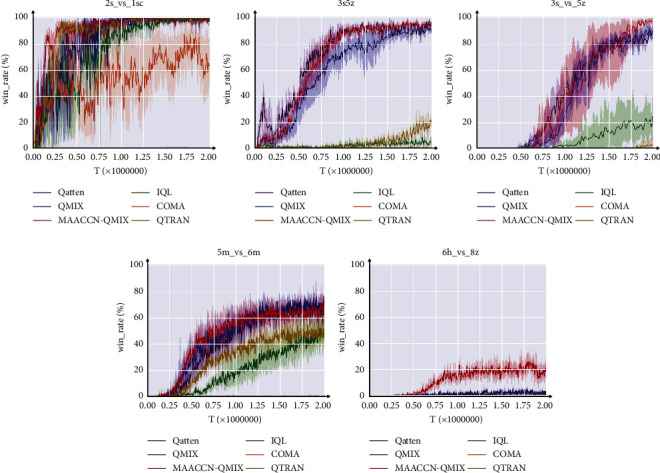
Experimental results of the MAACCN-QMIX algorithm on SMAC.

We conducted a comparative experiment in five scenarios, where complicated scenarios necessitated more effective cooperative tactics. As depicted in Figure 3, we add the MAACCN structure to the QMIX algorithm. QMIX is currently recognized as an algorithm with excellent performance, which employs a mix network to nonlinearly combine local agent functions, and QMIX has good scalability [10, 26], which adopts the paradigm of centralized training and decentralized execution. MAACCN-QMIX is a method for multiagent attentional communication with the common network, while QMIX and IQL are the baseline algorithms. In the majority of situations, our MAACCN-QMIX algorithm provides significantly enhanced performance over the baseline method. In simple scenario 3s5z and hard scenario 3s_vs_5z, the MAACCN-QMIX algorithm produces faster convergence and superior final outcomes than the baseline method. In extremely challenging scenario 6h_vs_8z, the existing baseline algorithms achieve outcomes close to zero, while the approach presented in this research achieves an average victory rate of almost 25%.

Table 2 shows the final performance of different algorithms during testing (the maximum median of all test results obtained in the last 250*k* steps of the training process). In the majority of scenarios, the results indicate that agents can cooperate more effectively and attain optimal performance by utilizing the MAACCN approach. The direct use of the global reward to update policies produces nonstationarity, which becomes more severe as the number of agents increases, resulting in a low win rate for IQL. The loose limitations in complex scenarios may reduce the accuracy of its updating, which lowers the QTRAN algorithm's performance.

### 5.3. Ablation

We focus on three aspects of experimental analysis in particular. The first is to remove the mixture network from the third module of the MAACCN structure and replace it with the VDN summation method, therefore minimizing the effect of the mix network on the experiment. The second is the ablation experiment of the communication module. One of the contrasted algorithms is the TarMAC-VDN algorithm, which removes the common network and preserves the multihead attention communication module, and the other is the CommNet-VDN method, which removes the module of the communication network and replaces it with the communication structure of the CommNet [11] algorithm. The MAACCN-without-DAgger algorithm abandons the DAGGER framework but keeps the common network. The final point to consider is the feasibility of using the common network as the expert network to guide the decisions of agents based solely on the DAgger framework.

**Figure 4:**
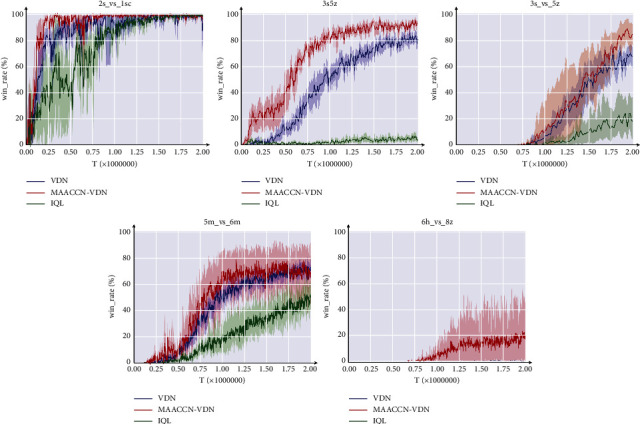
Experimental results of the MAACCN-VDN algorithm on SMAC.

Figure 4 shows the results of comparing the MAACCN-VDN algorithm and the baseline method in three different scenarios. In all scenarios, the convergence rate is demonstrably superior to alternative baseline algorithms, and the performance is superior as well. In scenario 3s5z, our MAACCN-VDN algorithm achieves a high win rate first and is markedly superior to the baseline method of VDN. This demonstrates that the MAACCN approach has a high degree of universality, allowing it to be used with other value-based algorithms.

**Figure 5:**
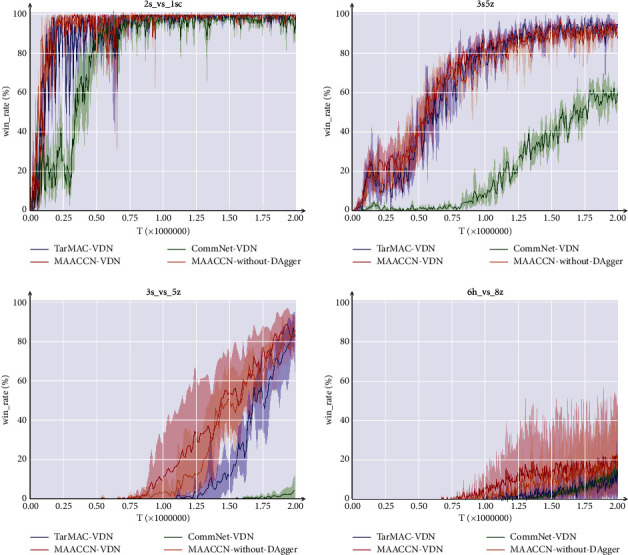
Ablation study of the communication composition.

Figure 5 verifies the validity of the communication mode in our algorithm. In all three scenarios, CommNet-VDN performs the poorest since an agent merely uses the action-observation sets of every agent and simply merges them. Both TarMAC-VDN and MAACCN-VDN perform better than CommNet-VDN, indicating that the hidden layer has richer information. Also, it demonstrates that the multihead attention communication structure described in this study is able to extract useful information from a complicated environment. The performance of the TarMAC-VDN algorithm can catch up with the MAACCN-VDN algorithm in scenario 3s5z, but not in other scenarios. Compared with TarMAC-VDN, the results of MAACCN-without-DAgger demonstrates that extracting consensus information from the common network as communication information is more beneficial than extracting communication information simply from the action-observation sets or the hidden layers of the current network, validating the efficacy of the common network module. The results of scenario 3s_vs_5z and scenario 6h_vs_8z show that the method of MAACCN-VDN outperforms the existing communication methods in terms of performance and convergence speed within the DAgger framework.

**Figure 6:**
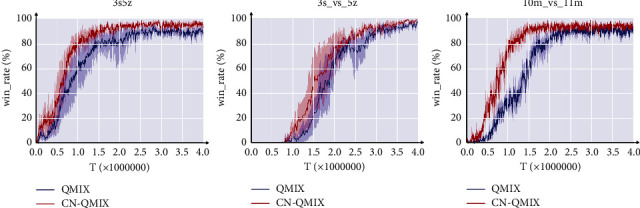
Experimental results of the CN-QMIX algorithm on SMAC.

We validate the impact of the common network within the DAgger framework. Based on QMIX, the CN-QMIX algorithm adds only the common network without any additional attention mechanisms or communication structures. Compared to benchmark algorithm QMIX, a significant performance disparity may be detected. As the number of training steps increases in Figure 6, the CN-QMIX algorithm tends to converge faster and become more stable. This clearly demonstrates the viability of the main hypothesis that the common network can be utilized as an expert to aid agents in making better judgements in big and complicated situations.

**Figure 7:**
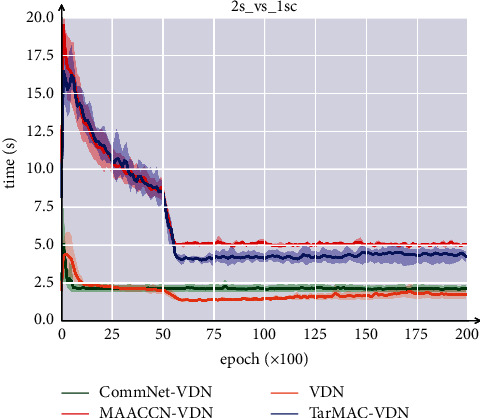
Experimental results of time cost for different algorithms.

Table 3 displays the GPU memory requirements of various algorithms. After each algorithm is trained over a period of time (epoch = 1000), the GPU memory consumption is obtained by calculating the mean several times. The ordinate of Figure 7 represents the average amount of time consumed per epoch by each algorithm. The experiment is conducted on scenario 2s_vs_1sc, as all algorithms can quickly identify the optimal strategy there. In order to minimize interference from other variables, each algorithm runs on a single GPU. The VDN algorithm has the smallest memory and time overhead, whereas the TarMAC-VDN algorithm with a communication module significantly increases memory and time overhead. Compared to the TarMAC-VDN algorithm, the MAACCN-VDN algorithm requires more memory due to the addition of a common network. Since the communication module is essential and the time cost of adding a common network is tolerable, we employ the MAACCN algorithm for better performance.

## 6. Conclusion

This paper proposes a consensus storage mechanism to model the consensus information of other agents by adding an additional common network to store the historically best-performing policy networks. The communication module is utilized to enhance the depiction of the entire network via a multihead attention mechanism. The MAACCN algorithm may fully utilize the knowledge of the common network, expanding the source of information while not limiting the information of the current policy network. In addition, under the DAgger framework, we regard the common network as an expert network to guide the policy of the agent, and the feasibility of this operation is verified through ablation experiments.

## Figures and Tables

**Algorithm 1:**
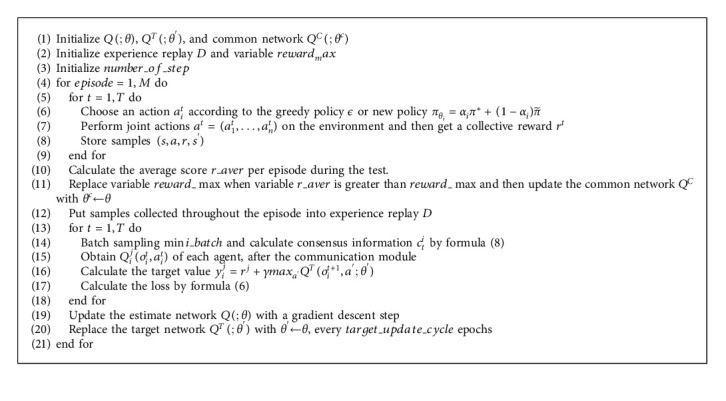
Multiagent attentional communication with the common network.

**Table 1:** Training parameters of the MAACCN algorithm.

Parameters	Value	Descriptions
Lr	0.0005	The learning rate
Epsilon	1	Probability of exploration
Min_epsilon	0.05	Minimum probability of exploration
Anneal_steps	50000	The annealing steps of exploration
T_max	2000000	The total step size of training
N_episodes	1	The number of episodes sampled at an epoch
Evaluate_cycle	100	The interval of the evaluation cycle
Evaluate_epoch	32	Frequency of evaluation
Batch_size	32	The batch data size for training
Buffer_size	5000	The size of the buffer
Target_update_cycle	200	The update interval of the target network
hidden_dim	64	The dimension of a hidden layer
Head	8	The number of the multihead

**Table 2:** Maximum median performance % of the algorithms tested.

Scenarios	MAACCN-QMIX	QMIX	VDN	IQL	Heuristic	QTRAN	Qatten	COMA
2s_vs_1sc	**100**	100	100	100	0	100	100	96
3s5z	**97**	91	87	9	42	20	95	0
5m_vs_6m	75	75	**78**	59	0	58	74	0
3s_vs_5z	**100**	97	73	46	0	15	97	0
6h_vs_8z	**30**	3	0	0	0	0	4	0

**Table 3:** Experimental results of GPU memory cost for different algorithms.

Scenes	MAACCN-VDN	TarMAC-VDN	CommNet-VDN	VDN
2s_vs_1sc	1490M	1120M	690M	680M
5m_vs_6m	1550M	1150M	710M	680M
3s_vs_5z	2010M	1480M	750M	730M

## Data Availability

Experiments were conducted on the StarCraft multiagent challenge (SMAC). Open source environment can be found in the paper “the StarCraft multiagent challenge.”

## Appendix

### A. Algorithm

The procedure of MAACCN which is a value-based algorithm.

### B. Parameters

Parameters required for MAACCN training are shown in Table 1. Choosing from the controller the action that corresponds to the maximum Q-value is the strategy of an agent. Multiple agents utilize their own techniques to interact with the environment, collect sample data from each episode into the experience replay, and then choose batches of data from the buffer to train agents. The agent generates and loads *N*_*episode* *s* sample data into the memory pool at each epoch. After receiving (*batch*, *episode* *s*_ *da* *ta*) data, an agent calculates the current Q-value at each moment of each batch, as well as the target Q-value for the next moment, depending on the value-based mode. The total loss is calculated by first summing the two portions of Q values for all agents, then calculating the loss of each batch of each episode at each instant *l*=(*Q* − (*r*+max_*a*_ *Q*^*target*^))^2^, and finally summing the loss of all episodes at all times. The loss was finally optimized using the RMSProp optimization approach.
